# Ethics issues identified by applicants and ethics experts in Horizon 2020 grant proposals

**DOI:** 10.12688/f1000research.52965.2

**Published:** 2021-09-06

**Authors:** Ivan Buljan, David G Pina, Ana Marušić

**Affiliations:** 1 Department of Research in Biomedicine and Health, University of Split School of Medicine, Split, 21000, Croatia; 2 European Research Executive Agency, European Commission, Brussels, 1049, Belgium

**Keywords:** research ethics, ethics review, grant assessment, Horizon 2020

## Abstract

**Background:** We assessed the ethics review of proposals selected for funding under the Marie Skłodowska-Curie Actions (MSCA) and the European Research Council (ERC) in Horizon 2020, EU’s framework programme for research and innovation, 2014-2020.

**Methods:** We analysed anonymized datasets for 3,054 MSCA individual fellowships (IF), 417 MSCA Innovative Training Networks (ITN), and 1,465 ERC main-listed proposals with ethics conditional clearance, over four years (2016 to 2019). The datasets included the information on ethics issues identified by applicants in their proposal and ethics issues and requirements identified by ethics experts during the ethics review.

**Results:** 42% of proposals received ethical clearance. For proposals with conditional ethics clearance (n=3546), most of the identified ethics issues by both applicants and ethics experts were in the ethics categories related to humans; protection of personal data; environment, health and safety; and non-EU countries. Ethics experts identified twice as many ethics issues compared to applicants across funding schemes, years, and from high- and low-research performing countries. ERC grants had the highest number of ethics requirements per proposal (median (Md)=8, interquartile range (IQR=4-14), compared to ITN (Md=6, IQR=3-13) and IF grants (Md=3, IQR=2-6). The majority of requirements had to be fulfilled after grant agreement: 99.4% for IF, 99.5% for ITN, and 26.0% for ERC. For 9% of the proposals, the requirements included the appointment of an independent ethics advisor and 1% of the proposals had to appoint an ethics advisory board.

**Conclusions:** Many applicants for highly competitive H2020 funding schemes lack awareness of ethics issues raised by their proposed research. There is a need for better training of researchers at all career stages about ethics issues in research, more support to researchers from research organizations to follow the funding agencies requirements, as well as further development and harmonization of the ethics appraisal process during grant assessment.

## Introduction

Ethics is a universal aspect of the research process, from planning and conduct of research, social and cultural relations in research, to reporting of research findings and use of research results. In recent years, ethics has received significant attention from not only researchers, but also policy makers, funders, and lay population due to the rising awareness of the importance of ethics issues in research and consequences of unethical behaviour in research ( ALLEA, 2017; Mejlgaard *et al.*, 2020). The existence of ethics standards in research leads to the greater cultural, social, and national equality in research by providing standardized approach to issues in different contexts ( Nordling, 2018), and compliance with ethics standards is expected from all who perform research. It is also an important part of education and training in research ( Resnik, 2020).

Researchers should be aware of ethics issues that arise from their research plan and take necessary steps to address these issues. However, evidence shows that researchers generally do not perform well in identifying ethics issues in their own research ( Taljaard *et al.*, 2011). We have even less evidence about how researchers identify and address ethics issues in grant proposals, mostly due to the lack of available data on this aspect of research grant evaluation, despite abundance of information and instructions by funding agencies ( European Commission, 2013). The evaluation of researcher’s ability to identify and address ethics issues in research proposals follows the same principles as scientific evaluation of grant proposals, so that the current gold standard in granting agencies is evaluation by ethics experts in a process of ethics review, which comes usually just after scientific evaluation of grant proposals ( European Commission, 2019a). It is expected that, if grant applicants are fully capable to detect ethics issues in research proposals, their assessments should not differ from that of ethics experts.

The aim of this study was to describe the ethics review process in two of the flagship funding programmes of Horizon 2020 (H2020) – the EU’s research and innovation framework programme from 2014 to 2020: the Marie Skłodowska-Curie Actions (MSCA) and the European Research Council (ERC). More specifically, we analysed the Individual Fellowships (IF) and Innovative Training Networks (ITN) from MSCA, and ERC grants (namely the Starting, Consolidator and Advanced grants). We compared ethics issues identified by the applicants in grant proposals with those identified by ethics experts during the ethics review, as well as ethics requirements resulting from this process.

## Methods and analytical framework

### The MSCA and ERC programme

The MSCA and ERC programmes are two flagships of H2020. Their initial earmarked budget amounted to 6.2 and 13.1 billion Euro, respectively, representing around 25% of the initial H2020 overall budget ( European Commission, 2013).

The MSCA programme is composed of a set of funding schemes (actions), mostly dedicated to research training and career development of mobile researchers, especially those at an earlier career stage ( Pina *et al.*, 2015). Among these actions, the IF covers transnational postdoctoral research training, and ITN specifically targets doctoral training of early-stage researchers. These two actions represent around 95% of the total number of MSCA proposals submitted and funded.

The ERC grants are funding principal investigators and their teams on frontier research. The programme has remained quite stable since its inception in 2007. Taken together, the MSCA IF, ITN, and the ERC grants represent more than 42% of the overall eligible proposals submitted to the whole H2020 programme, and with success rates in the range of around 8–15%, they are among the most competitive funding scheme of the framework programme.

Both MSCA and ERC programmes cover all fields of research, in a so-called bottom-up approach. This means that applicants are free to apply for any type of research, with no *ex-ante* priority given to specific topics. This contrasts with most of the rest of the H2020 programmes. However, for evaluation purposes, MSCA applicants are asked to submit proposals in one of eight scientific panels: Chemistry (CHE), Social Sciences and Humanities (SOC), Economic Sciences (ECO), Information Science and Engineering (ENG), Environment and Geosciences (ENV), Life Sciences (LIF), Mathematics (MAT), and Physics (PHY). In the ERC, proposals are submitted to scientific panels belonging to one of the three following research domains: Life Sciences (LS), Physical Sciences and Engineering (PH), and Social Sciences and Humanities (SH). To facilitate the comparison of the two programmes, we grouped the ECO and SOC panels of MSCA as a proxy for the SH domain of ERC, and the CHE, ENG, ENV, MAT, PHY panels of MSCA as a proxy for the PH domain of ERC. Only the LIF panel in MSCA was considered as an equivalent of the LS domain of ERC.

### Ethics review procedure

**Figure 1.**
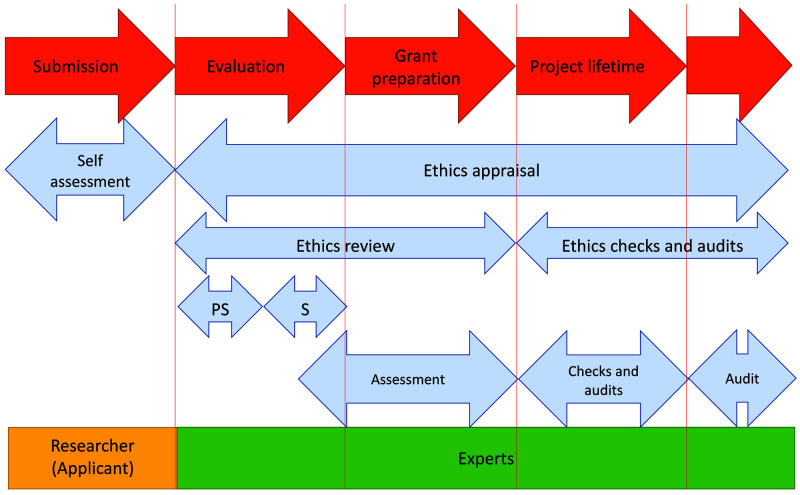
Ethics appraisal procedure in Horizon 2020. PS – pre-screening, S – screening. Pre-screening in European Research Council (ERC) evaluation process is performed by internal ERC staff.

The ethics review procedure applies systematically for all proposals considered for funding (i.e. proposals ranked in the main list for funding) ( Figure 1). It usually follows the scientific evaluation, where expert reviewers assess and score proposals based on a set of three criteria for the MSCA (Excellence, Impact, and Implementation) and the sole criterion of Excellence for ERC. All submitted proposals contain an ethics self-assessment checklist filled in by the applicants, where they list, in their views and opinion, the ethics issues raised by their proposed research ( European Commission, 2019a). The applicants must submit an Ethics Annex only in the case if they declare any ethics issue in the checklist.

Proposals that do not raise immediate ethics issues are generally pre-screened by ERC dedicated internal staff, to assess if a full screening is necessary. In MSCA, main-listed proposals are in most of the cases sent automatically for screening. The following step in the ethics review process, the ethics screening, is performed by two or more external independent experts and, in some cases, it is followed by an ethics assessment when a more in-depth analysis is needed for serious or complex ethics issues raised by the proposed research. Grant proposals involving research with human embryonic stem cells or human embryos automatically go for ethics assessment ( European Commission, 2019a). Ethics experts are involved in both the screening and the assessment phase. For ERC proposals, ethics assessments are managed internally, whereas MSCA ethics assessments are conducted by the Ethics Sector within the Directorate-General for Research and Innovation (DG RTD).

The final output of the ethics review is the Ethics Summary Report (EthSR), drafted by ethics experts, which includes an ethics opinion on the proposal. This opinion can have the following outcome: i) No ethics issues, when the ethics review confirms that the proposal does not raise any ethics issue; ii) Ethics clearance, when ethics issues are adequately addressed in the proposals, and there is no need for change upon signature of the grant agreement; and iii) Conditional ethics clearance, when the clearance is subject to conditions, usually in the form of “ethics requirements” listed in the EthSR. Ethics requirements must be implemented either before the grant signature, by updating the ethics section of the project description, or at a specific time after the grant signature in the form of ethics deliverables. When an ethics assessment is needed for proposals raising serious or complex ethics issues, the applicants are usually requested to submit additional information for an in-depth ethics analysis before the ethics experts formulate their opinion. Proposals conditionally cleared can therefore be the result of an ethics screening or an ethics assessment. In very rare cases, a project proposal can be rejected on ethics grounds ( European Commission, 2019a)

### Data sources and description of ethics issues categories

We analysed the data for main-listed proposals from MSCA IF, MSCA ITN calls in 2016-2019, and for ERC calls in 2017-2019, the year for which the data were available in the electronic Submission and Evaluation of Proposals (SEP) tool of the European Commission. SEP integrated the full ethics evaluation process in 2016 for MSCA and in 2017 for ERC. When this study was initiated in 2020, the ethics evaluation process was still not fully completed for IF and ERC, so we considered only the data up to the 2019 calls. As our datasets were based on the structured information available in the EthSR from SEP, they are partially incomplete. They did not include MSCA proposals for which an ethics assessment was performed by the Ethics Sector from DG RTD. These proposals include the cases with human embryonic stem cells or human embryos. The proposals that did not raise any ethics issues or were cleared by the ethics experts were not analysed further, as they correspond to the cases where the applicants either addressed correctly the ethics issues in their proposals or the proposed research did not raise any ethics issues. We focused on the main-listed proposals that were conditionally cleared, as they correspond to the cases where differences existed between what was declared by the applicants and what was later flagged by ethics experts.

The data from SEP were split into two datasets, the first containing the information about the ethics issues identified by applicants and ethics experts, and the second one containing the information about ethics requirements listed by ethics experts. For both datasets, we collected data about the proposal call year, scientific panel/domain, the host country (of the coordinating institution), and ethics issues/requirements categories. We also had the information related to ethics checks. The list of ethics issues as categorized by the European Commission is available in the Extended data ( Buljan *et al.*, 2021). The ethics issues are divided into 12 distinct categories, as listed in the Ethics Self-Assessment Checklist: *Human embryos/foetuses; Humans; Human cells/tissues; Personal data; Animals; Third countries; Environment, Health & safety; Dual use; Exclusive focus on civil applications; Misuse*; and *Other ethics issues* ( European Commission, 2019a).

For ethics requirements dataset, there was no *Exclusive focus on civil applications* category, and the category *General* was merged with the *Other* category.

### Ethical considerations

We worked on anonymized datasets, without insight about the actual content of the proposal, or the names of the applicants or ethics experts, so that issues of personal data protection were not applicable.

### Data analysis

Ethics issues and requirements are presented as frequencies and percentages, by type of action (for MSCA) or grant (ERC), call year, and scientific domain. Ethics issues identified by the applicants and ethics experts are presented separately. The number of ethics issues/requirements per proposal is presented as median, with interquartile range (IQR). We calculated odds ratios for the probability that ethics issues in a specific category is identified by ethics experts vs. that it is identified by applicants.

We performed linear regression analysis, where the criterion was the number of ethics issues in a proposal identified by ethics experts, and with the type of action, year, host country, and number of issues in a specific category in a proposal identified by applicants as potential predictors. Host countries ware categorized into research low- and high-performing categories, based on a previously reported composite indicator ( Pina *et al.*, 2019). Research high-performing countries were Austria, Belgium, Denmark, Finland, France, Germany, Israel, the Netherlands, Norway, Sweden, Switzerland, and the United Kingdom, and low-performing countries were Bulgaria, Croatia, Cyprus, Czech Republic, Estonia, Greece, Spain, Hungary, Ireland, Iceland, Italy, Lithuania, Luxemburg, Latvia, Malta, Poland, Portugal, Romania, Serbia, Slovenia, Slovakia, and Turkey.

To determine the characteristics of main-listed proposals subject to ethics review, we collected the information about call year, type of action, number of requirements per proposal and research domain in logistic regression. For this analysis, we recoded ethics category groups by omitting *Human embryos/foetus* category due to small sample size and collapsing *Dual use; Misuse*; *Other, Exclusive use on civil applications* (linear regression only) and *General* (logistic regression only) categories into *Other* category.

The results are presented as odds ratios with 95% confidence intervals (CI) and Nagelkerke R squared. All analyses were performed using the R software environment for statistical computing and graphics ( R Core Team, 2020).

## Results

The total number of main listed proposals in the period from 2016-2019 for MSCA and 2017-2019 for ERC, was 485 proposals from ITN, 5365 proposals from IF, and 2632 proposals from ERC (Table S1 in Extended data ( Buljan *et al.*, 2021)). Of these, we identified 4936 proposals (58%) that received conditional ethics clearance during the ethics review (excluding those from MSCA that went for an ethics assessment by DG RTD). This means that the remaining 3546 (42%) proposals either had no ethics issues or received ethics clearance ( Figure 1, Table S1 in Extended data ( Buljan *et al.*, 2021)). Proposals from individual granting schemes more often received ethics clearance (44.3% for ERC proposals and 43.1% for IF proposals) than proposals from research consortia (14.1% for ITN proposals).

### Ethics issues identified by applicants and ethics experts in conditionally cleared main-listed proposals

**Figure 2.**
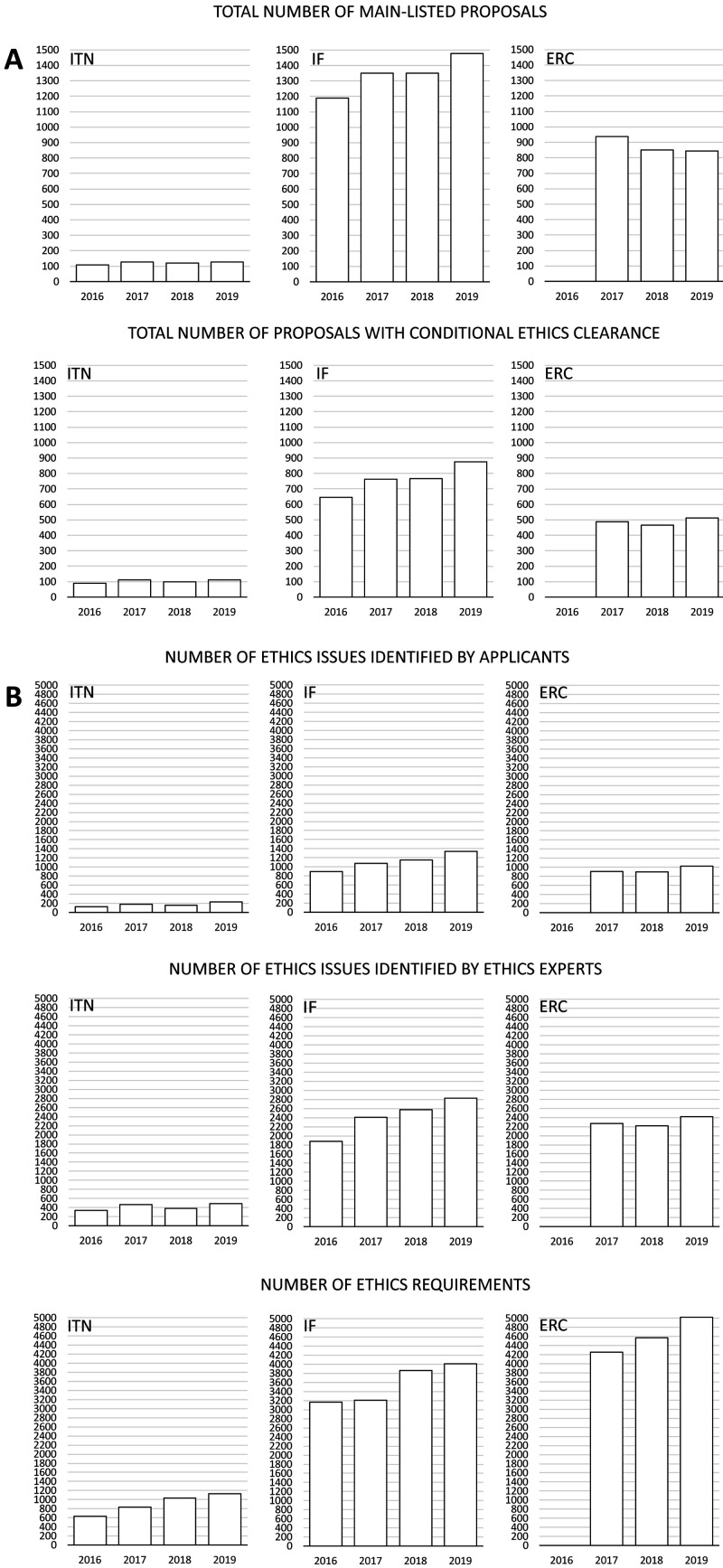
Distribution of the number of proposals, ethics issues and ethics requirements among different grant programmes from 2016 to 2019. ITN – Innovative Training Networks, IF – Individual Fellowships, ERC – European Research Council grants. The data are from years available in the SEP tool of the European Commission.

Applicants identified 707 ethics issues in 417 proposals from ITN (85.9% out of all main-listed proposals), 4469 issues in 3054 proposals from IF (56.9%), and 2826 issues in 1465 proposals from ERC (55.7%) ( Figure 2, Table S1 in Extended data ( Buljan *et al.*, 2021)). Ethics experts identified more than two times more issues than applicants: 1676 for ITN, 9695 for IF, and 6912 for ERC proposals. This trend was stable across different types of grants and call years ( Figure 2).

**Figure 3.**
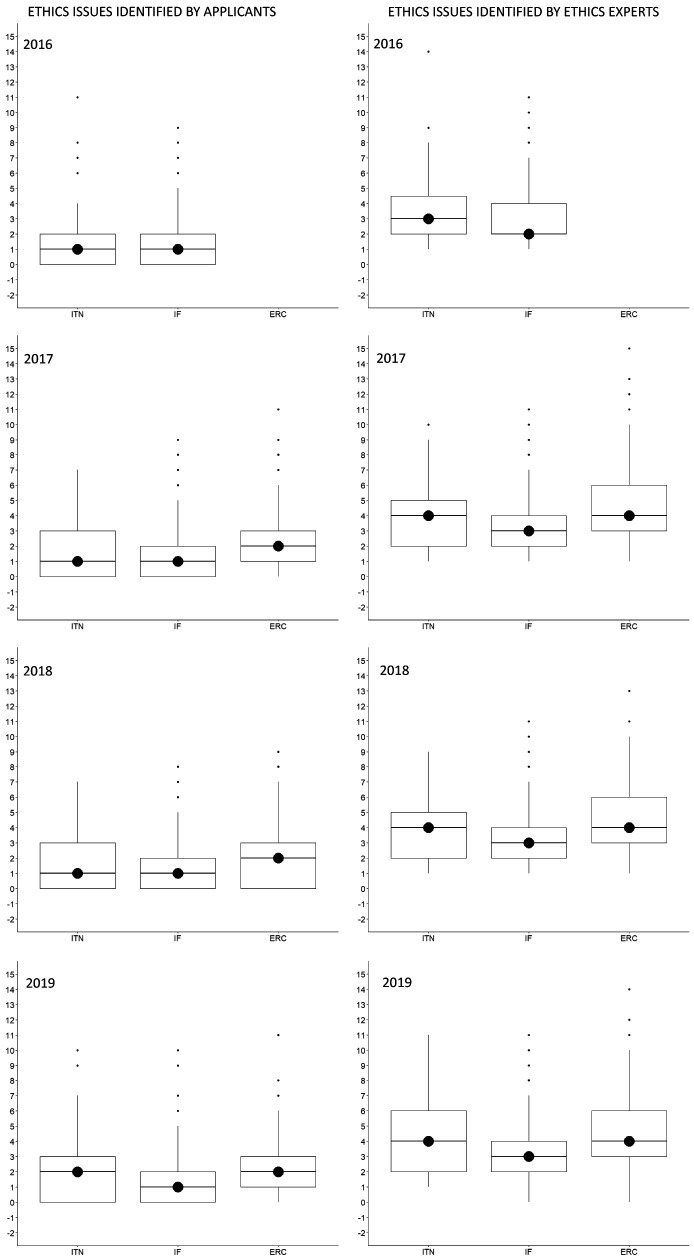
Median number of ethics issues per proposal identified by applicants and by ethics experts, 2016-2019. ITN – Innovative Training Networks, IF – Individual Fellowships, ERC – European Research Council grants. The data are from years available in the SEP tool of the European Commission.

The median (Md) number of ethics issues identified by an applicant per proposal was 1 (IQR=0-3) for ITN, 1 (IQR=0-2) for IF, and 2 (IQR=1-3) for ERC, whereas ethics experts identified a median of 4 ethics issues (IQR=2-5) for ITN, 3 (IQR=2-4) for IF, and 4 (3-6) for ERC. This was true for all call years ( Figure 3). The most frequently identified ethics issues categories overall were *Humans*; *Protection of personal data; Animals*; and *Non-EU countries* (Table S1 in Extended data ( Buljan *et al.*, 2021)). Applicants had higher odds to identify ethics issues related to *Humans*, *Human cells/tissues*, *Protection of personal data*, and *Animals* categories, whereas ethics experts were more likely to identify ethics issues in categories *Non-EU countries* and *Environment, health and safety* (Table S1 in Extended data ( Buljan *et al.*, 2021)).

**Figure 4.**
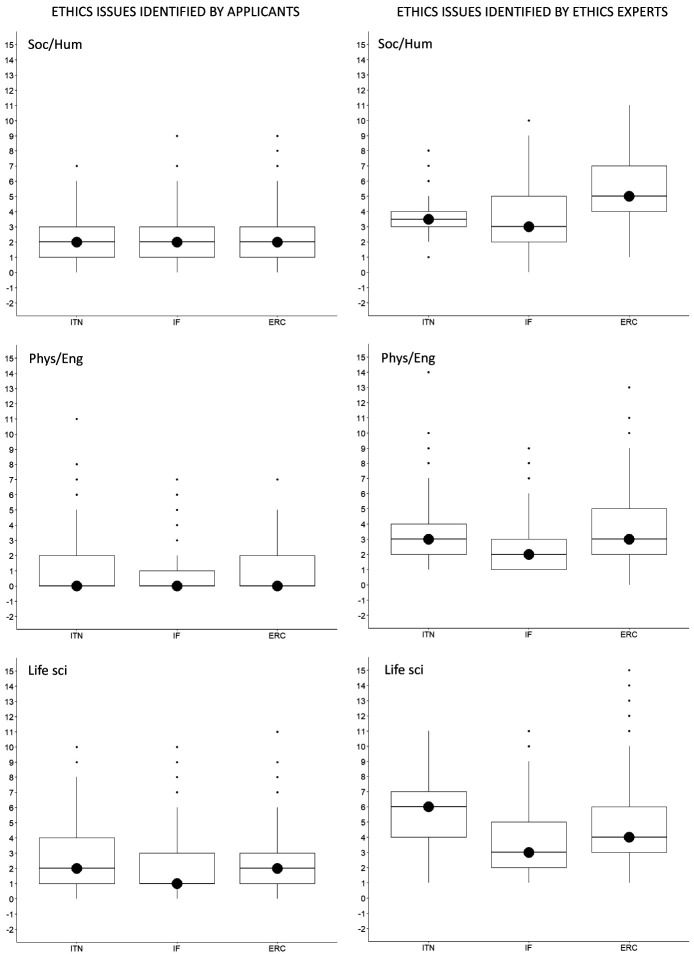
Median number of ethics issues per proposal identified by applicants and by ethics experts, by research domain. ITN – Innovative Training Networks, IF – Individual Fellowships, ERC – European Research Council grants. The data are from years available in the SEP tool of the European Commission. Soc/Hum – Social Sciences and Humanities, Phys/Eng – Physical Sciences and Engineering, Life sci – Life Sciences.

With regard to the research domain, applicants from Life Sciences identified the most issues, with 3719 ethics issues (n=3719), followed by Social Sciences and Humanities (n=2916) and Physical Sciences and Engineering (n=1367). On the other side, ethics experts identified two times more ethics issues for proposals in Life Sciences (n=7652) and Social Sciences and Humanities (n=6055), and three times more ethics issues in Physical Sciences and Engineering (n=4576) (Table S2 in Extended data ( Buljan *et al.*, 2021)). The same was found for the median number of ethics issues per proposal ( Figure 4).

**Figure 5.**
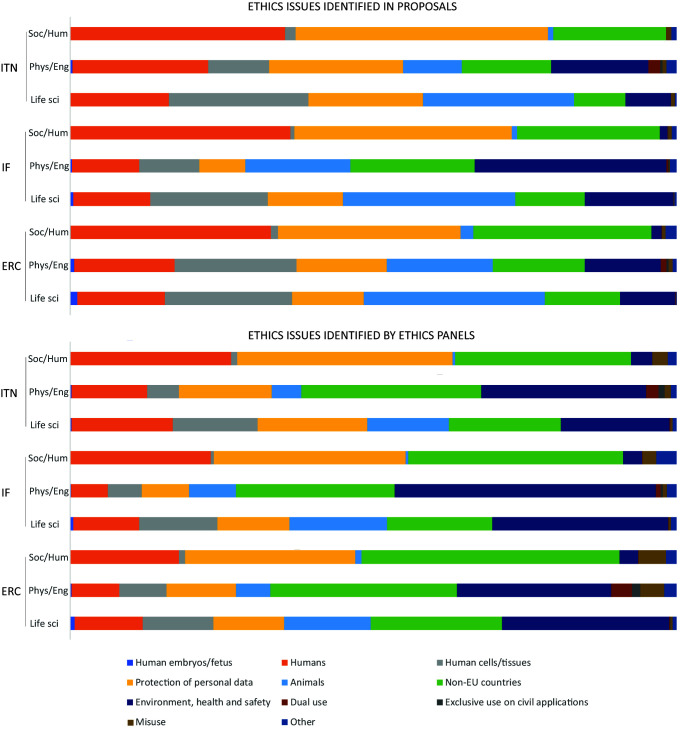
Distribution of ethics issues categories identified by proposal applicants and ethics experts, by research domain. ITN – Innovative Training Networks, IF – Individual Fellowships, ERC – European Research Council grants. The data are from years available in the SEP tool of the European Commission. Soc/Hum – Social Sciences and Humanities, Phys/Eng – Physical Sciences and Engineering, Life sci – Life Sciences.

**Figure 6.**
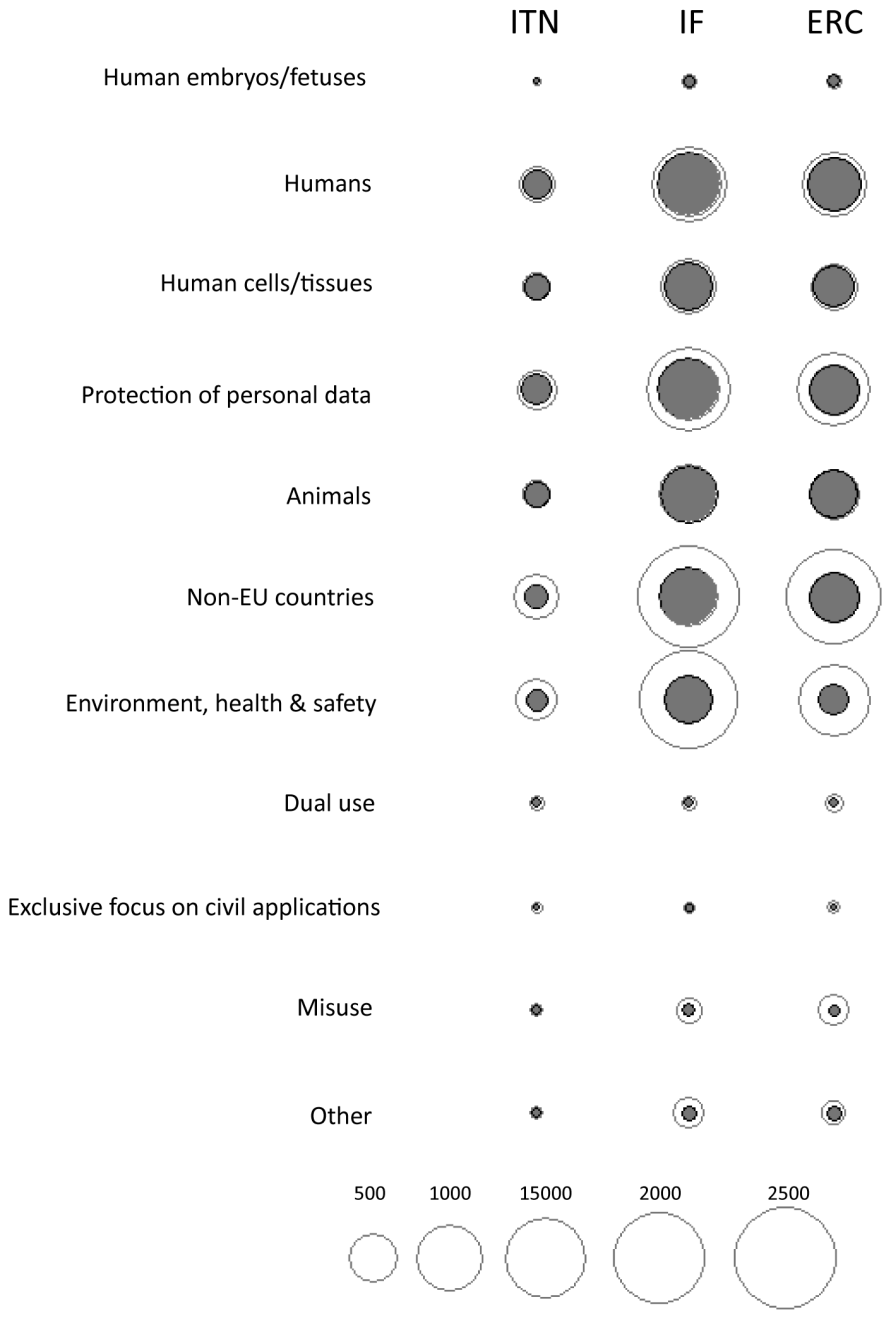
Comparison between ethics issues identified by proposal applicants (grey) and ethics experts (white), by ethics categories. ITN – Innovative Training Networks, IF – Individual Fellowships, ERC – European Research Council grants. The data are from years available in the SEP tool of the European Commission.

For Social Sciences and Humanities, the most prevalent categories were *Humans*, *Protection of personal data* and *Non-EU countries*, whereas in Life Sciences and Physical Sciences and Engineering, *Animals*, *Human cells/tissues*, and *Environment, health and safety* categories were more often identified by both the applicants and ethics experts ( Figure 5). When observing the total number of ethics issues by category, the above six categories comprised most of the identified ethics issues, regardless of the type of grant ( Figure 6). The greatest discrepancy in absolute numbers between project applicants and ethics experts was for the *Non-EU countries* and *Environment, health and safety* categories ( Figure 6). Proposals from the Physical Sciences and Engineering were characterized by the highest number of issues related to Dual (military) use, identified by both the applicants and the experts ( Figure 6).

Regression analysis, where we assessed which proposal characteristics predicted higher number of identified ethics issues per ethics category by ethics experts, showed that it was more likely that the proposal will have significantly more ethics issues in the same category, identified by ethics experts when it was an ERC proposal, coming either from Life Sciences or Social Sciences and Humanities, or a newer proposal, as well as if it had issues related to the *Protection of personal data*, *Non-EU countries*, and *Environment, health and safety* categories, and had multiple issues identified by applicants in the same ethics category (Table S3 in Extended data ( Buljan *et al.*, 2021)). On the other hand, lower number of ethics issues in a same category identified by ethics experts were related to the proposals from MSCA funding schemes (both ITN and IF), from Physical Sciences and Engineering, from an earlier call, and had issues related to the *Human cells/tissues*, *Animals* or *Other* categories, as well as fewer ethics issues by ethics category identified by applicants (Table S3 in Extended data ( Buljan *et al.*, 2021)). However, the best predictor for the number of ethics issues identified by ethics experts was the number of ethics issues by ethics category identified by applicants, and it explained 19% of the variance of the criteria when independently entered in linear regression. Proposals which had multiple identified ethics issues per ethics category were more likely to have more ethics issues in a specific category identified by ethics experts.

### Ethics requirements

**Figure 7.**
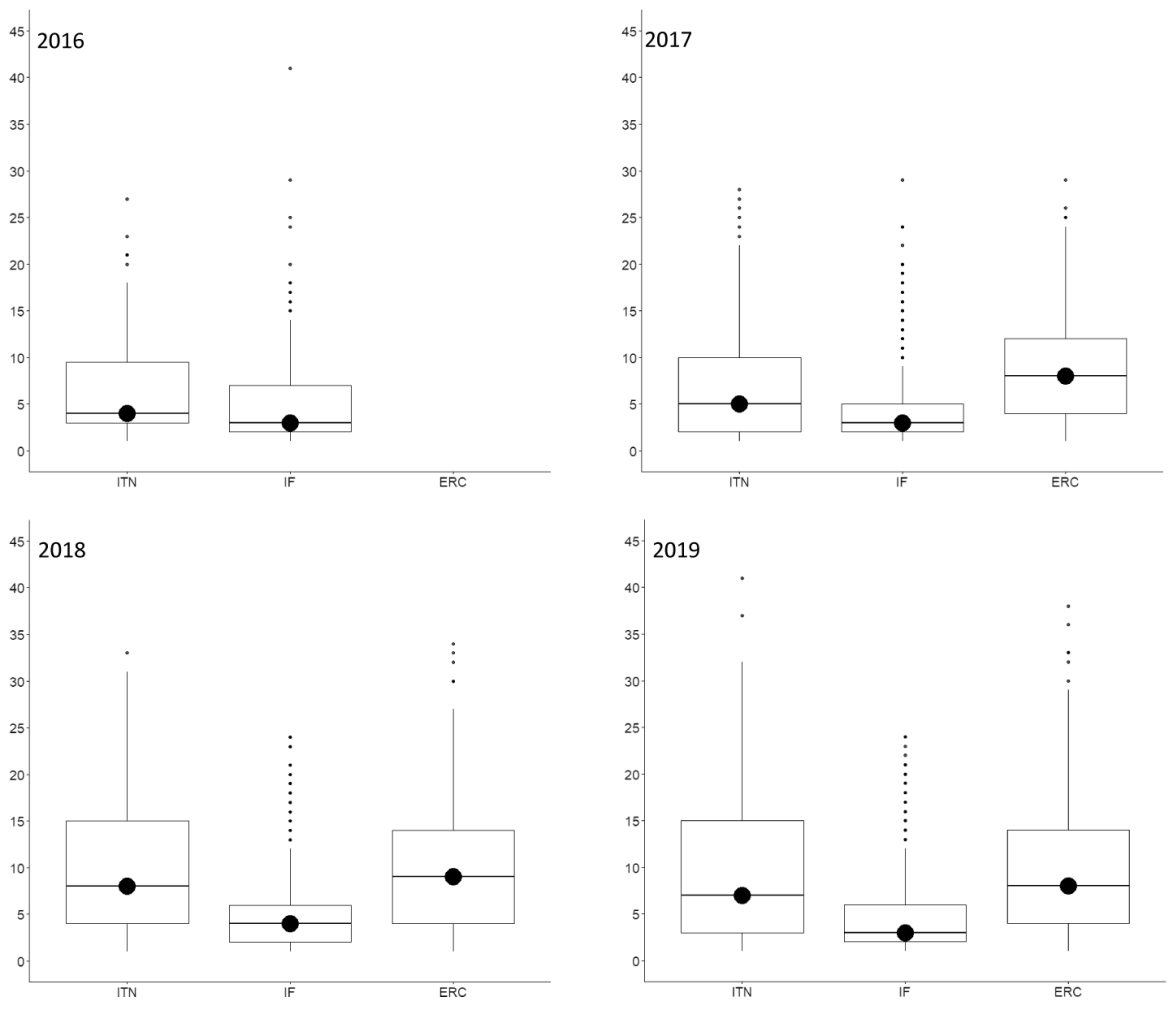
Median number of ethics requirements per proposal, 2016-2019. ITN – Innovative Training Networks, IF – Individual Fellowships, ERC – European Research Council grants. The data are from years available in the SEP tool of the European Commission.

**Figure 8.**
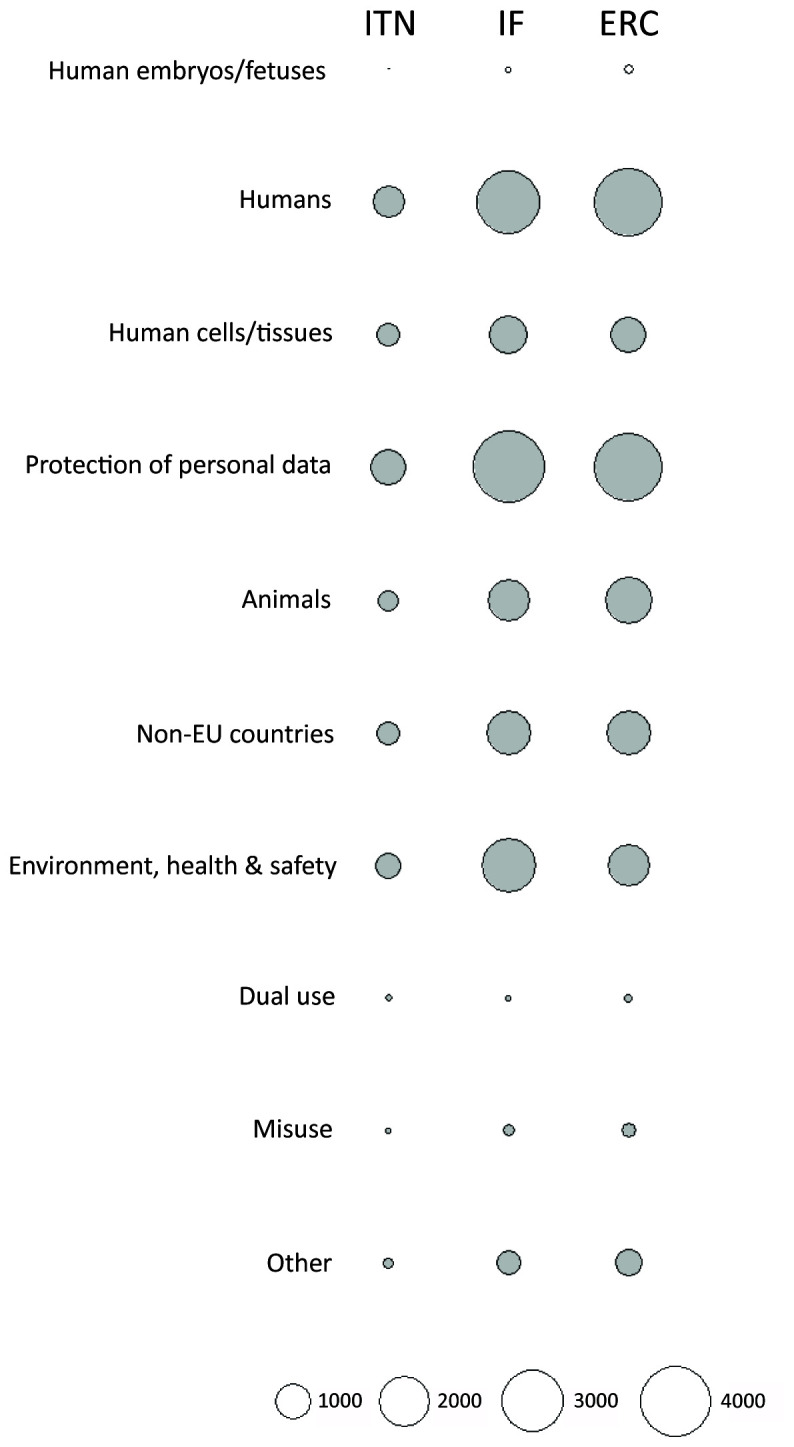
Ethics requirements identified by ethics experts, by ethics category. ITN – Innovative Training Networks, IF – Individual Fellowships, ERC – European Research Council grants. The data are from years available in the SEP tool of the European Commission.

**Figure 9.**
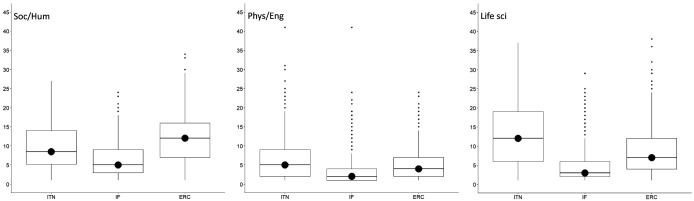
Median number of ethics requirements identified by ethics experts, by research domain. ITN – Innovative Training Networks, IF – Individual Fellowships, ERC – European Research Council grants. The data are from years available in the SEP tool of the European Commission. Soc/Hum – Social Sciences and Humanities, Phys/Eng – Physical Sciences and Engineering, Life sci – Life Sciences.

**Figure 10.**
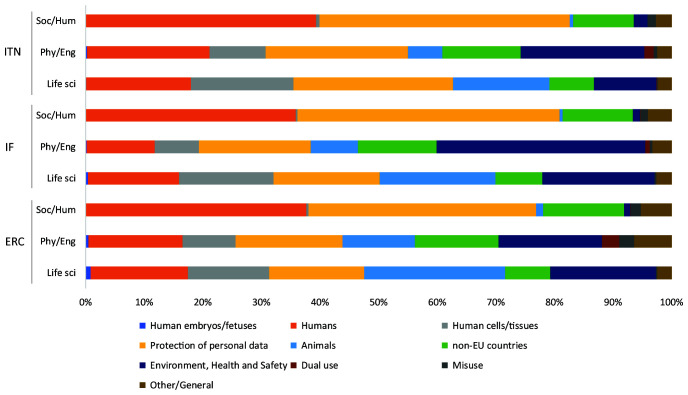
Distribution of ethics requirements identified by ethics experts, by research domain. ITN – Innovative Training Networks, IF – Individual Fellowships, ERC – European Research Council grants. The data are from years available in the SEP tool of the European Commission. Soc/Hum – Social Sciences and Humanities, Phys/Eng – Physical Sciences and Engineering, Life sci – Life Sciences.

The number of ethics requirements was greater than the number of ethics issues identified by ethics experts (3625 for ITN, 14249 for IF and 13846 for ERC) ( Figure 2). ERC had the highest median number of ethics requirements per proposal (Md=8, IQR=4-14), compared to ITN (Md=6, IQR=3-13) and IF (Md=3, IQR=2-6) ( Figure 7). The greatest proportion of ethics requirements was in the *Humans, Human Cells/Tissues*, *Non-EU countries,* and *Environment, health and safety* categories ( Figure 8; Table S4 in Extended data ( Buljan *et al.*, 2021)). Proposals from Life Sciences and Social Sciences and Humanities domains had the greatest number of requirements (n=12544 and n=12377, respectively). There were 6799 requirements for proposals in Physical Sciences and Engineering (Table S5 in Extended data ( Buljan *et al.*, 2021)). All three types of grants were similar in the number of ethics requirements per proposal across domains ( Figure 9). Although proposals from Life Sciences and Social Sciences and Humanities had a similar total number of requirements, most of the ethics requirements for Social Sciences and Humanities were in the *Humans* and *Protection of personal data* categories. Requirements for proposals in Life Sciences were linked to all ethics categories ( Figure 10).

The majority of requirements’ deliverables were scheduled to be fulfilled after the grant agreement signature: 14162 (99.4%) for IF; 3606 (99.5%) for ITN, and 3600 (26.0%) for ERC. The median deliverable’s month of implementation, when a requirement had to be fulfilled, was month 3 for IF (IQR=1-5, range=1-36), month 12 for ITN (IQR=12-12, range=1-48), and month 12 (IQR=12-12, range=0-55) for ERC.

Ethics checks were required for 43 ITN proposals (10.2%), 141 IF proposals (4.6%), and 185 ERC proposals (12.6%). The predictors of whether a proposal will involve an ethics check were a higher number of ethics requirements, being an MSCA ITN proposal, coming from Social Sciences and Humanities, and being a proposal from an older call year (Table S6 in Extended data ( Buljan *et al.*, 2021)). It was also more likely that a proposal will go for an ethics check if it had multiple ethics requirements in the collapsed *Other* category, and less likely if the multiple requirements were for *Animals* or *Environment, health and safety* categories (Table S6 in Extended data ( Buljan *et al.*, 2021)).

Ethics experts can require the appointment of an independent ethics advisor or an ethics advisory board, for proposals with complex or serious ethics issues, or when they consider that the applicants did not demonstrate sufficient proficiency in identifying or managing ethics issues. 6.2% of all conditionally cleared proposals had such a requirement (5.3% for ethics advisor and 0.9% for ethics advisory boards). ERC proposals had more such requirements: 14.9% proposals needed ethics advisor vs 1.2% of MSCA proposals, and 2.5% needed ethics advisory boards vs 0.3% for MSCA proposals. For MSCA proposals, there was no clear difference, except for the predominance of proposals from Social Sciences and Humanities among proposals needing an ethics advisor (8 out of 11 proposals) and Life Science proposals among ITN (12 out of 32 proposals). Half (51.1%) of the proposals that required some kind of ethics monitoring, either by ethics advisors or boards, during the project lifetime also required an ethics check.

## Discussion

To the best of our knowledge, this is the first study that explored in detail the characteristics of the ethics review in highly competitive H2020 proposals across research disciplines and funding schemes. We showed that about half (42%) of the proposals did not require an ethics review, either because the proposed research did not raise ethics issues or the applicants adequately addressed ethics issues. However, for those that received conditional ethics clearance, both the individual researchers (in IF and ERC granting schemes) and research consortia (ITN grants) were not good in identifying ethics issues generated by the proposed research because ethics experts identified two times more ethics issues. The applicants were better at identifying “classical” ethics issues – those related to research involving humans or human cells and tissues, protection of personal data, and animals, but not as good in identifying ethical challenges related to the research involving collaboration with other countries, and environmental, health and safety issues. The reason for this may be the fact that the latter ethics categories are more legal in nature, requiring different types of legal documentation and procedures ( European Commission, 2019b), and may not be perceived as a “true” ethics issue. The same was true for ethics categories that were generally rarely identified: *Dual-use* and *Exclusive focus on civilian applications* (research that could be used for military applications) and *Misuse* (research for unethical purposes such as terrorism). Ethics experts, who are considered as the “golden standard” for ethics review, identified twice as more ethics issues, in general and per proposal. For some proposals, they required formal ethical support and monitoring in the form of individual ethics advisors or ethics advisory board or as ethics checks at some points during the life time of the project.

We observed important differences in identified ethics issues between different research domains and grant types. The proposals with fewer ethics issues in the same category identified by ethics experts were those where applicants identified fewer issues in the same category, in MSCA compared to ERC grant calls, those from Physical sciences and Engineering domain, proposals from earlier periods/years and those with ethics issues in categories *Human cells/tissues*, *Animals* or *Other* (including *Dual use, Misuse, General* and *Other ethics issues*). There was general congruence between the applicants and ethics experts in identification of ethics issues categories, except that experts identified more issues. The finding that ERC proposals had more ethics issues than MSCA ITN and IF may be related to the ERC selection priority for breakthrough research, which brings higher risk and uncertainty ( European Research Council, 2019). Proposals from Life Sciences and Social Sciences and Humanities had more ethics issues and resulting ethics requirements than those from Physical Sciences and Engineering because they more often include research with human participants or with animals. Finally, very small but significant influence of the year of proposal was found for both the number of issues and ethics requirements, with higher numbers in later years. The study time period (2016–2019) was the period of significant change for one of the major ethics issues: in 2016, the General Data Protection Regulation (GDPR) was enacted in the EU, with the implementation starting from mid-2018 ( European Commission, 2016). This means that the researchers in later grant years could become more aware of these new requirements and paid more attention to ethical issues in general. However, the *Personal data protection* category did not emerge as a predictor in any of the regression analyses in our study. It is also possible that the efforts that the European Commission and research performing or research granting institutions have invested in the promotion responsible conduct of research ( Ščepanović *et al.*, 2021; Mejlgaard *et al.*, 2020) had an effect over the years. However, the median number of ethics issues per proposal remained stable over the study period, implying that applicants did not improve their performance in identifying ethics issues over time. Longer follow-up studies and controlled study designs, such as interrupted time series ( Pina *et al.*, 2021), are needed to test the effect of time on ethics appraisal of grant proposals.

The need for more proactive approach to ethics training and assistance to researchers is clear. For example, applicants from the Physical Sciences and Engineering domain may be, in many cases, unaware of ethics issues raising from the development of new prototypes, instruments or software involving any testing with human participants. This need is also in the number of proposals where the ethics experts required the appointment of an Ethics Advisor, in order to help in the management and monitoring of the ethics issues during the project. In some other proposals, the establishment of a larger Ethics Advisory Board was deemed necessary to coordinate the management of complex and/or serious ethics issues. Such projects very rarely included ethics monitoring already in the proposal, meaning that the applicants and their institutions were not aware of the complexities or seriousness of ethics issues in their research.

The complexity of ethics issues raised by proposed research differed between different grant calls. While individual fellowship (IF) proposals required an ethics check during the ethics review process in about 5% of the cases, ITN proposals, which are submitted by a consortium of research institutions, and ERC grants, which are individual proposals but involving high-risk research, required ethics checks two times more often. Taking ethics check requirement as a proxy for complex and specific ethics issues, regression analysis identified that such proposals had more ethics requirements and were coming from the Social Sciences and Humanities Domain, had more ethics requirements in the *Non-EU* and *Dual use, Misuse, General* and *Other* categories and from older calls. This complexity is also reflected in the number of proposals that had to fulfil ethics requirements during the project lifetime (around 99% for IF and ITN and 26% for ERC). ERC proposals also had almost ten times more requirements for an ethics advisor than MSCA grants. The reasons for these differences are not clear – they may be related to the specificities of the calls or proposed research, or the organization of the ethics appraisal process. Our study showed clear differences between the ethics review procedure between ERC and MSCA, but we currently do not have any evidence on relevant outcomes that would indicate possible differences in their effectiveness.

Our study had some limitations that need to be acknowledged. We did not have information about the actual content of the proposals so that the ethics issues could be assessed independently. We considered ethics experts review procedure as the golden standard in ethics assessment, just as scientific expert review is used as a measure of the scientific quality of the proposal ( Pina *et al.*, 2021). The sample in our study included only the highest ranked proposals considered for funding, as only these proposals undergo ethics review. We do not know whether the applicants whose proposals did not get funded had better, worse or similar skills in identifying ethics issues as those of funding proposals. We also did not evaluate the proposals that received ethics clearance, so we did not determine whether the reason for ethical clearance of half of the main-listed projects was because the applicants adequately addressed ethics issues or if it was because the project did not raise ethics issues. The results of our study could be considered as an indication of best ethics practices among participants in EU grant proposals, as they select the best proposals in highly competitive calls. Finally, we did not explore in detail the qualitative (textual) content of the ethics review, which could identify in specific ethics issues raised by the proposed research and those addressed in ethics requirements.

It is difficult to put the results from our study in a more general context as there are no studies of ethics as an important aspect of grant evaluation ( Buljan *et al.*, 2020). We know that authors of research papers are generally poor in reporting ethics issues ( Taljaard *et al.*, 2011). There are many studies assessing different aspects of publications ethics, including examples such as consent for publishing identifiable medical images ( Roguljić *et al.*, 2020) or authorship ( Marušić *et al.*, 2011), because the data for such studies are publicly available, in contrast to the information on grant evaluation.

Research performing and research funding organizations are the places where the problem of the competence of researchers to identify and address ethics issues must be addressed. This problem is a part of a greater problem of shaping structures and processes for research ethics and research integrity to promote responsible conduct of research ( Mejlgaard *et al.*, 2020). Different approaches to these issues have been described, ranging from education and training in research ethics ( Dubois *et al.*, 2008) and research integrity ( Marušić *et al.*, 2016). For example, some organizations ( LARI, 2021) provide ethics and integrity support to early-stage researchers in the form of integrity coaches or advisors, which help researchers navigate through ethics and integrity requirements during their research. There are also efforts at the policy level ( ALLEA, 2017), as well as at the level of organizational culture and climate for responsible research ( Viđak *et al.,* 2021), but we do not have quality evidence of whether these efforts actually work.

It is also important to keep in mind the differences between research disciplines, which have been already recognized in the context of ethics codes ( Aagaard-Hansen & Johansen, 2008). In our study, research in different disciplines did not result in the difference in the number of identified ethics issues, but in the different pattern of the distribution among ethics categories. Research proposals from Physical Sciences and Engineering had mostly ethics issues related to collaboration with non-EU countries and those related to environment, health and safety. They also had the largest prevalence of ethics issues in *Dual use* category, compared to other domains. While the proposals from Life Sciences did not have a clear predominance among major ethics issues – those related to research involving humans and animals, non-EU countries and health and environment, dominating ethics issues in the proposals from Social Sciences and Humanities were related to the involvement of human participants and personal data protection. Researchers from Social Sciences and Humanities were not good at identifying these issues, as the difference in ethics issues identified by applicants and the issues and requirements put forward by ethics experts was greatest for proposals in this research domain. This has been recognized by the European scientific community and specific research ethics guidance has been developed for social sciences ( European Commission, 2018).

Finally, the heterogeneity of research ethics and research integrity structures and processes in Europe ( European Commission, 2019b), as well as differences in research integrity codes across Europe ( Desmond & Dierickx, 2021), do not help researchers in understanding what is expected from them in regard to research ethics, especially when they work in collaborations that cross country boundaries. As we did not find differences between countries that are high performers in research and those that are not, the problems in identifying ethics issues in their research proposals seem to be common to all research communities across Europe.

## Conclusion

Our study demonstrated that researchers are not always adequately competent in recognizing and addressing ethics issues in research that they propose to grant agencies, and that this problem persisted over the last four years of EU research funding framework. Although there are intensive efforts to promote training and restructuring of research ethics and research integrity framework at all levels of research in Europe ( Mejlgaard *et al.,* 2020), more concrete actions that are actually resulting in a positive change are needed. We also need to study the process of ethics evaluation of grant proposals and research project execution in order to identify critical points for change and/or improvement, as well as facilitators and barriers to successful management of ethics issues in research. Although such studies may be methodologically complex and demanding, and involve both quantitative and qualitative approaches, they are needed so that we can better understand the problem and formulate solutions. Only when we have evidence for effective actions, we will be able to provide support to researchers to deal with ethical issues in their research and prepare for emerging ethical challenges, such as the use of gene-editing ( European Group on Ethics in Science and New Technology, 2021) and artificial intelligence ( European Parliament, 2019) in research.

## Data availability

### Underlying data

OSF: Ethics issues identified by applicants and ethics experts in Horizon 2020 grant proposals. https://doi.org/10.17605/OSF.IO/T765V ( Buljan *et al.*, 2021).

This project contains the following underlying data:

- Data file 1. Datasets.xlsx

### Extended data

OSF: Ethics issues identified by applicants and ethics experts in Horizon 2020 grant proposals. https://doi.org/10.17605/OSF.IO/T765V ( Buljan *et al.*, 2021).

This project contains the following extended data:

- List of ethics issues as categorized by the European Commission
- Table S1. Number of ethical issues identified by applicants and ethics experts for MSCA ITN and IF and the ERC, per call year
- Table S2. Number of ethical issues identified by applicants and ethics experts, by scientific domain, for MSCA ITN and IF and the ERC
- Table S3. Linear regression for predicting number of ethics issues per category in a proposal identified by ethics experts
- Table S4. Number of ethics requirements identified by applicants and ethics experts in MSCAITN and IF and the ERC, per call year
- Table S5. Number of ethics requirements, by scientific domain, for the ERC
- Table S6. Logistic regression for prediction of whether a proposal will go to ethics check (0-No,1-Yes)

### Reporting guidelines

OSF: STROBE checklist for ‘Ethics issues identified by applicants and ethics experts in Horizon 2020 grant proposals’. https://doi.org/10.17605/OSF.IO/T765V ( Buljan *et al.*, 2021).

Data are available under the terms of the Creative Commons Zero "No rights reserved" data waiver (CC0 1.0 Public domain dedication).

### Disclaimer

All views expressed in this description are strictly those of the authors and may in no circumstances be regarded as an official position of the European Research Executive Agency or the European Commission.

## References

[ref-1] Aagaard-Hansen J Johansen MV: Research ethics across disciplines. *Anthropology Today.* 2008;24(3):15–19. 10.1111/j.1467-8322.2008.00585.x

[ref-4] Buljan I Marusic A Pina D: Ethics issues identified by applicants and ethics experts in Horizon 2020 grant proposals: Supplementary files.2021. https://osf.io/cyp4w/10.12688/f1000research.52965.1 PMC8356263 34394917

[ref-3] Buljan I Pina D Marušić A: Meta Research: Ethics assessment of H2020 Marie Skłodowska-Curie individual fellowships.PEERE Conference, Valencia, Spain, 29 September–1 October2020. Reference Source

[ref-5] Desmond H Dierickx K: Research integrity codes of conduct in Europe: Understanding the divergences. *Bioethics.* 2021;35(5):414–428. 10.1111/bioe.12851 33550603

[ref-6] DuBois JM Dueker JM Anderson EE: The development and assessment of an NIH-funded research ethics training program. *Acad Med.* 2008;83(6):596–603. 10.1097/ACM.0b013e3181723095 18520469 PMC4474180

[ref-7] European Commission: Regulation (EU) No 1291/2013 of the European Parliament and of the Council of 11 December 2013 establishing Horizon 2020 - the Framework Programme for Research and Innovation (2014-2020) and repealing Decision No 1982/2006/EC Text with EEA relevance.2013; viewed 15 April 2021. Reference Source

[ref-8] European Commission: Regulation (EU) 2016/679 of the European Parliament and of the Council of 27 April 2016 on the protection of natural persons with regard to the processing of personal data and on the free movement of such data, and repealing Directive 95/46/EC (General Data Protection Regulation).2016; viewed 12 March 2021. Reference Source

[ref-9] European Commission: Ethics in Social Science and Humanities.2018; viewed 12 March 2021. Reference Source

[ref-10] European Commission: Horizon 2020 Guidance: How to complete your ethics self-assessment.2019a; viewed 20 October 2020. Reference Source

[ref-11] European Commission: Mutual Learning Exercise (MLE) on Research Integrity: Final Report.2019b; viewed 12 March 2021. 10.2777/72096

[ref-2] European Federation of Academies of Sciences and Humanities (ALLEA): The European Code of Conduct for Research Integrity.2017; Accessed 17 August 2020. Reference Source

[ref-12] European Group on Ethics in Science and New Technology: Ethics of Genome Editing.2021; viewed 15 April 2021. Reference Source

[ref-13] European Parliament: EU guidelines on ethics in artificial intelligence: Context and implementation.2019; viewed on 12 March 2021. Reference Source

[ref-14] European Research Council: Information for Applicants to the Starting and Consolidator Grant 2020 Calls.2019; viewed 12 March 2021. Reference Source

[ref-15] Luxembourg Agency for Research Integrity (LARI). viewed 15 April 2021. Reference Source

[ref-16] Marušić A Bošnjak L Jerončić A: A systematic review of research on the meaning, ethics and practices of authorship across scholarly disciplines. *PLoS One.* 2011;6(9):e23477. 10.1371/journal.pone.0023477 21931600 PMC3169533

[ref-17] Marusic A Wager E Utrobicic A: Interventions to prevent misconduct and promote integrity in research and publication. *Cochrane Database Syst Rev.* 2016;4(4):MR00038. 10.1002/14651858.MR000038.pub2 27040721 PMC7149854

[ref-18] Mejlgaard N Bouter LM Gaskell G: Research integrity: nine ways to move from talk to walk. *Nature.* 2020;586(7829):358–360. 10.1038/d41586-020-02847-8 33041342

[ref-19] Nordling L: Europe's biggest research fund cracks down on 'ethics dumping'. *Nature.* 2018;559(7712):17–18. 10.1038/d41586-018-05616-w 29970912

[ref-20] Pina DG Barać L Buljan I: Effects of seniority, gender and geography on the bibliometric output and collaboration networks of European Research Council (ERC) grant recipients. *PLoS One.* 2019;14(2):e0212286. 10.1371/journal.pone.0212286 30763395 PMC6375614

[ref-21] Pina DG Buljan I Hren D: A retrospective analysis of the peer review of more than 75,000 Marie Curie proposals between 2007 and 2018. *eLife.* 2021;10:e59338. 10.7554/eLife.59338 33439120 PMC7806263

[ref-22] Pina DG Hren D Marušić A: Peer Review Evaluation Process of Marie Curie Actions under EU's Seventh Framework Programme for Research. *PLoS One.* 2015;10(6):e0130753. 10.1371/journal.pone.0130753 26126111 PMC4488366

[ref-30] R Core Team: R: A language and environment for statistical computing.2021; viewed 20 Spril 2021. Reference Source

[ref-23] Resnik DB: What Is Ethics in Research & Why Is It Important?2020; viewed 18 January 2021. Reference Source

[ref-24] Roguljić M Buljan I Veček N: Deidentification of facial photographs: a survey of editorial policies and practices. *J Med Ethics.* 2020; medethics-2019-105823. 10.1136/medethics-2019-105823 32253363

[ref-25] Ščepanović R Labib K Buljan I: Practices for Research Integrity Promotion in Research Performing Organisations and Research Funding Organisations: A Scoping Review. *Sci Eng Ethics.* 2021;27(1):4. 10.1007/s11948-021-00281-1 33502638 PMC7840650

[ref-26] Taljaard M McRae AD Weijer C: Inadequate reporting of research ethics review and informed consent in cluster randomised trials: review of random sample of published trials. *BMJ.* 2011;342:d2496. 10.1136/bmj.d2496 21562003 PMC3092521

[ref-27] Viđak M Barać L Tokalić R: Interventions for organizational climate and culture in academia: A scoping review. *Sci Eng Ethics.* 2021;27(2):24. 10.1007/s11948-021-00298-6 33783667

