# Supplementary material for: Ethics issues identified by applicants and ethics experts in Horizon 2020 grant proposals
Source: F1000Res. 2021 Sep 6;10:471. Originally published 2021 Jun 15. [Version 2] doi: 10.12688/f1000research.52965.2 (PMC8356263; doi:10.12688/f1000research.52965.2)
Supplement: Peer review history

# Peer review: Reviewer response for version 2

**DOI:** 10.5256/f1000research.77142.r93573

**Published:** 2021-09-07

**Authors:** Vasiliki Mollaki

**Affiliations:** 1 National Commission for Bioethics and Technoethics, Athens, Greece

I have no further comments to make.

Is the work clearly and accurately presented and does it cite the current literature?

Yes

If applicable, is the statistical analysis and its interpretation appropriate?

Yes

Are all the source data underlying the results available to ensure full reproducibility?

Yes

Is the study design appropriate and is the work technically sound?

Yes

Are the conclusions drawn adequately supported by the results?

Yes

Are sufficient details of methods and analysis provided to allow replication by others?

Yes

Reviewer Expertise:

Genetics, Ethics, Bioethics

I confirm that I have read this submission and believe that I have an appropriate level of expertise to confirm that it is of an acceptable scientific standard.

# Peer review: Reviewer response for version 1

**DOI:** 10.5256/f1000research.56302.r90648

**Published:** 2021-08-10

**Authors:** Juraj Koppel

**Affiliations:** 1 Institute of Animal Physiology, Centre of Biosciences, Slovak Academy of Sciences, Košice, Slovakia; 2 Presidium of Slovak Academy of Sciences, Bratislava, Slovakia

This is an interesting article summarizing important data concerning ethics review processes in MSCA and ERC grants. The manuscript is well presented, the design is fully appropriate and adequate details are given. The discussion section is well elaborated, conclusions are clear and fully adequate. Indeed, the reviewer acknowledges that this is a unique study exploring in details the ethics review in H2020 grant proposals across research disciplines and funding schemes.

 Few minor comments and suggestions:

1. “All submitted proposals contain an ethics self-assessment checklist filled in by the applicants, where they list, in their views and opinion, the ethics issues raised by their proposed research ( European Commission, 2019a). The applicants must explain in the proposal how they will address and manage these ethics issues over the course of their projects, if funded.“ In fact, applicants have to submit an (ideally explanatory) Ethics Annex only in the case if they declare any ethics issue in the checklist.
2. “All analyses were performed using the R programming software v.4.0.3 ( R Core Team, 2020).“ Perhaps a better description is "the R software environment for statistical computing and graphics“.
3. “proposals from older years“ could be rephrased as "proposals from earlier periods/years".
4. In the context of number of ethics issues frequently seen in grant proposals the statement “The need for more proactive approach to ethics training and assistance to researchers“ is really crucial and has to be underlined. For illustration – applicants e.g. in ENG or PHY focused on the development of new instruments or software are in general fully unaware that any testing involving humans raises ethics issues.

Is the work clearly and accurately presented and does it cite the current literature?

Yes

If applicable, is the statistical analysis and its interpretation appropriate?

Yes

Are all the source data underlying the results available to ensure full reproducibility?

Yes

Is the study design appropriate and is the work technically sound?

Yes

Are the conclusions drawn adequately supported by the results?

Yes

Are sufficient details of methods and analysis provided to allow replication by others?

Yes

Reviewer Expertise:

developmental physiology, bioethics, research integrity, science management

I confirm that I have read this submission and believe that I have an appropriate level of expertise to confirm that it is of an acceptable scientific standard.

# Response

**Published:** 2021-08-27

**Authors:** Ana Marušić

**Affiliations:** University of Split School of Medicine, Croatia

Thank you for the comments, we made all suggested changes in the revised manuscript.

# Peer review: Reviewer response for version 1

**DOI:** 10.5256/f1000research.56302.r87517

**Published:** 2021-07-26

**Authors:** Vasiliki Mollaki

**Affiliations:** 1 National Commission for Bioethics and Technoethics, Athens, Greece

The manuscript aims to assess the ethics review of proposals selected for funding under specific H2020-funded programmes between 2016-2019. It is found that ethics experts identified twice as many ethics issues compared to applicants across funding schemes, years, and from high- and low-research performing countries, indicating the need to train researchers in identifying and addressing ethics issues raised by their research.

 The manuscript is well structured and the methodology is clearly presented.

**Minor comments:**

- **Section The MSCA and ERC programme:** "Among those actions" should be "Among these actions".
- **Section Ethics review procedure: **"which include an ethics opinion on the proposal" should be "which includes an ethics opinion on the proposal".
- **Section Data sources and description of ethics issues categories:** "Those proposals include the" should be " These proposals include the".
   
  - "The ethics issues are divided into 11 distinct categories" should be "The ethics issues are divided into 12 distinct categories".
- **Section Data analysis: **"and number issues in a specific category" should be "and number of issues in a specific category".
- **Section Results: **"had no ethics issues or received ethical clearance" should be "had no ethics issues or received ethics clearance".
- **Section Ethics issues identified by applicants and ethics experts in conditionally cleared main-listed proposals: **"That trend was stable across" should be " This trend was stable across".
- **Figure 5 and 10:** Label "Human embryos/fetus" should be "Human embryos/ fetuses".
- **Figure 5 and 6:** Label "exclusive use on civil applications" should be "exclusive focus on civil applications".
- **Section Discussion:** "with human and animal participants" do you mean "with human participants and animals"?
- **Section Conclusion:** "Although such studies may me methodologically" should be "Although such studies may be methodologically".

Is the work clearly and accurately presented and does it cite the current literature?

Yes

If applicable, is the statistical analysis and its interpretation appropriate?

Yes

Are all the source data underlying the results available to ensure full reproducibility?

Yes

Is the study design appropriate and is the work technically sound?

Yes

Are the conclusions drawn adequately supported by the results?

Yes

Are sufficient details of methods and analysis provided to allow replication by others?

Yes

Reviewer Expertise:

Genetics, Ethics, Bioethics

I confirm that I have read this submission and believe that I have an appropriate level of expertise to confirm that it is of an acceptable scientific standard.

# Response

**Published:** 2021-08-27

**Authors:** Ana Marušić

**Affiliations:** University of Split School of Medicine, Croatia

Thank you for the comments - we made all suggested changes in the revised manuscript
